# Estimating the Level of Carbamoylated Plasma Non-High-Density Lipoproteins Using Infrared Spectroscopy

**DOI:** 10.3390/jcm8060774

**Published:** 2019-05-31

**Authors:** Sigurd E. Delanghe, Sander De Bruyne, Linde De Baene, Wim Van Biesen, Marijn M. Speeckaert, Joris R. Delanghe

**Affiliations:** 1 Department of Nephrology, Ghent University Hospital, 9000 Ghent, Belgium; Sigurd.Delanghe@ugent.be (S.E.D.); Wim.Vanbiesen@ugent.be (W.V.B.); Marijn.Speeckaert@ugent.be (M.M.S.); 2 Department of Clinical Chemistry, Ghent University Hospital, 9000 Ghent, Belgium; Sander.Debruyne@uzgent.be (S.D.B.); Linde.Debaene@ugent.be (L.D.B.); 3 Research Foundation-Flanders (FWO), 1000 Brussels, Belgium

**Keywords:** carbamoylation, chronic kidney disease, lipoproteins, infrared spectroscopy

## Abstract

Background: The increased cardiovascular morbidity and mortality observed in chronic kidney disease (CKD) patients can be partly explained by the presence of carbamoylated lipoproteins. Lipid profiles can be determined with infrared spectroscopy. In this paper, the effects of carbamoylation on spectral changes of non-high-density lipoproteins (non-HDL) were studied. Methods: In the present study, fasting serum samples were obtained from 84 CKD patients (CKD stage 3–5: *n* = 37 and CKD stage 5d (hemodialysis): *n* = 47) and from 45 healthy subjects. In vitro carbamoylation of serum lipoproteins from healthy subjects was performed using increasing concentrations of potassium cyanate. Lipoprotein-containing pellets were isolated by precipitation of non-HDL. The amount of carbamoylated serum non-HDL was estimated using attenuated total reflectance-Fourier transform infrared (ATR-FTIR) spectroscopy, followed by soft independent modelling by class analogy analysis. Results: Carbamoylation resulted in a small increase of the amide I band (1714–1589 cm^−1^) of the infrared spectroscopy (IR) spectrum. A significant difference in the amide II/amide I area under the curves (AUC) ratio was observed between healthy subjects and CKD patients, as well as between the two CKD groups (non-dialysis versus hemodialysis patients). Conclusions: ATR-FTIR spectroscopy can be considered as a novel method to detect non-HDL carbamoylation.

## 1. Introduction

Carbamoylation is a post-translational modification, playing a role in chronic kidney disease (CKD), comparable to the role of glycation in diabetes mellitus [1,2]. This non-enzymatic reaction is characterized by a covalent binding of isocyanic acid to the amino group (either the epsilon amino group of lysine residus or the N-terminal amino group) of amino acids, polypetides, and (lipo)proteins. This post-translational molecular modification contributes to the molecular ageing of proteins. Isocyanic acid is formed continuously in an equilibrium reaction with urea or by myeloperoxidase (MPO). MPO is an enzyme (present in e.g., neutrophils, monocytes, and some tissue macrophages) that catalyzes the oxidation of thiocyanate in the presence of hydrogen peroxide, producing isocyanate at inflammation sites (e.g., atherosclerotic plaques) [3]. Plasma isocyanic acid concentrations increase with a declining kidney function [4].

An increased cardiovascular morbidity and mortality (10–30 times higher than in the general population) has been reported in the end-stage renal disease (ESRD) population [5], which can be partly attributed to the influence of carbamoylated lipoproteins [6]. In renal failure, dyslipidemia contributes to a worsening kidney function. Proteinuria is accompanied by a marked elevation of low-density lipoproteins (LDL) [7]. Due to its carbamoylation, LDL can exert its prothrombotic [8] and atherosclerosis-prone effects by stimulating an increased adhesion of monocytes to endothelial cells [9], by inducing endothelial dysfunction [6] and endothelial mitotic cell death [10], and by promoting smooth muscle proliferation [11]. Carbamoylated LDL (cLDL) has been identified as the most abundant modified LDL isoform in human blood, which is also present in healthy individuals [12]. It is generated by carbamoylation of apolipoprotein B, the protein component of the LDL particle [13]. CKD is also associated with decreased serum high-density lipoprotein (HDL) concentrations. Carbamoylation of HDL leads to a loss of the atheroprotective function of HDL, illustrated by an impaired ability to promote cholesterol efflux from macrophages [14].

As carbamoylation is of major clinical importance, practical biomarkers for assessing carbamoylation and lipoprotein carbamoylation, in particular, are needed. In the present study, we explored the possibilities of infrared (IR) spectroscopy to assess non-HDL carbamoylation. The advantages of IR spectroscopy to determine lipid profiles were already applied in the past [15]. In this paper, the effects of in vitro carbamoylation on spectral changes of non-HDL were studied. Furthermore, non-HDL carbamoylation was investigated in healthy subjects, in non-dialysis (CKD stage 3–5) as well as in hemodialysis patients (CKD stage 5d).

## 2. Materials and Methods

### 2.1. Study Participants

The control group consisted of 45 healthy subjects (median age: 28 years, interquartile range (IQR): 24–33 years), whereas the patient group consisted of 84 CKD patients (CKD stage 3–5: *n* = 37, median age: 70 years, IQR: 56–75 years, and CKD stage 5d (hemodialysis): *n* = 47, median age: 67 years, IQR: 56–75 years) of the Department of Nephrology, Ghent University Hospital. The approval of this study was granted by the Ethical committee of the Ghent University Hospital (EC/2015/0932).

### 2.2. In Vitro Carbamoylation of Lipids

In vitro carbamoylation of lipids was achieved by adding increasing volumes of 0.5 mol/L potassium cyanate (KOCN) solution (Sigma–Aldrich, St. Louis, MO, USA) in a phosphate buffered salt (PBS) solution (0.1 mol/L, pH 8.0) (Sigma–Aldrich, MO, USA) to 1000 µL serum of healthy subjects. Serum samples were carbamoylated using increasing concentrations of KOCN: 0 mmol/L, 20 mmol/L, 50 mmol/L, 80 mmol/L and 100 mmol/L. In vitro carbamoylation was carried out for 48 h at 37 °C (these reaction conditions warrant a completeness of the reaction). Proof of carbamoylation was obtained by verifying the electrophoretic mobility of lipoprotein fractions on a lipoprotein agarose electrophosis (Hydragel 7, Sebia, Lisses, France) using a semi-automated HYDRASYS instrument (Sebia, Lisses, France). The separated lipoproteins were stained with a lipid-specific Sudan black stain. The excess of stain was removed with an alcoholic solution. The resulting electropherogram was evaluated visually.

### 2.3. In Vitro Oxidation

Oxidative stress is involved in the exacerbation of disease burden in CKD patients. In vitro oxidation of serum was performed to reveal potential influences on the infrared spectrum. Ten samples from the serum pool at the laboratory of clinical biology of the University Hospital in Ghent were randomly selected. One milliliter of each sample was pooled. Before the oxidation process, non-HDL were precipitated 5 times. According to a modified version of the method used by Coffey et al. [16], oxidized non-HDLs were prepared by dialyzing 4 mL of the serum pool against 400 mL isotonic saline (0.15 mol/L NaCl (VWR International, Haasrode, Belgium) dissolved in distilled water) containing 60 µmol/L CuSO4 (copper(II)sulphate pentahydrate, Merck Eurolab, Leuven, Belgium) during one hour. The dialysate was then changed to isotonic saline containing 0.5 mmol/L EDTA (BDH Chemicals, Poole, England) and dialysis continued during one hour with changes of dialysate every 15 min. After the oxidation process, non-HDL were precipitated 5 times from the oxidized serum pool.

### 2.4. Precipitation Procedure

All serum samples were centrifugated during 10 min at 3000× *g*. A precipitation reaction was performed, in which non-HDL fats very-low-density lipoprotein (VLDL), intermediate-density lipoprotein (IDL), lipoprotein(a), LDL and chylomicrons) were precipitated. 20 µL of a 13 mmol/L sodium phosphotungstate hydrate solution (Sigma Aldrich St Louis, MO, USA) and 5 µL of 2 mol/L MgCl_2_ (E. Merck KG, Darmstadt, Germany) were added to 200 µL serum. After vortexing, the samples were centrifuged (10 min, 6000× *g*) (centrifuge 54515 D, Eppendorf, Hamburg, Germany) [17]. The precipitate was subsequently dried for 48 h in an incubator. Completeness of the precipitation reaction was assessed by lipid electrophoresis of the serum pre- and post-precipitation (5 samples were precipitated in triplicate). The formed pellet was ground prior to analysis with attenuated total reflectance-Fourier transform infrared (ATR-FTIR) spectroscopy.

### 2.5. ATR-FTIR Analysis

All spectra were obtained by a Perkin Elmer Two ATR-FTIR spectrometer with the ATR accesory and spectrum 10 software (Perkin Elmer, Waltham, MA, USA). Before and after each analysis, the 50 mm ZnSE crystal was thoroughly cleaned with an alcoholic solution (Dax alcoliquid, Dialex biomedica, Sweden). A background scan was taken after the complete evaporation of the alcoholic solution. The lipoprotein powder was placed in contact with the surface of the crystal until complete covering. The pressure applied to the sample was standardised at 100 gauche to obtain a good contact between the sample and the crystal.

Three peaks were investigated: the carbonyl peak, the peak of the amide I band and the peak of the amide II band. Within-run coefficients of variation (CV) and between-run CV were calculated. The area under the curves (AUC) of the amide I and amide II bands were obtained by auto-labeling of the peaks in the Perkin Elmer 10 software.

Spectra were analyzed using the software program SIMCA version 14.1 (Umetrics, Sartorius Stedim Biotech, Umeå, Sweden). SIMCA (soft independent modeling of class analogy) was used to identify the spectral changes due to carbamoylation of lipoproteins. By applying various spectral filters, the noise was eliminated and the region of interest was selected. Data were normalized using the standard normal variate (SNV) method and were converted to their second derivative. The Savitsky-Golay algorithm allowed smoothing of the spectrum.

### 2.6. Routine Laboratory Measurements

After overnight fasting, blood samples were collected and centrifuged (10 min, 3000× *g*). Urea, creatinine, albumin, triglycerides, total and HDL-cholesterol concentrations were assayed using commercial reagents on a Cobas 8000 analyzer (Roche, Mannheim, Germany) [18]. The serum concentration of apolipoprotein B was determined by immunonephelometry on a Behring BN II nephelometer (Siemens, Marburg, Germany) [19]. The LDL-cholesterol concentration was estimated using the Friedewald-formula [20]. The estimated glomerular filtration rate (eGFR) was calculated with the Chronic Kidney Disease Epidemiology Collaboration (CKD-EPI) formula [21].

### 2.7. Statistics

Statistical analyses were performed using MedCalc (MedCalc, Mariakerke, Belgium). Normality of distributions was tested using the D’Agostino Pearson test. Data are expressed as median ± IQR or mean ± standard deviation (SD). Differences between patient groups were assessed using the Student’s *t*-test and the Kruskall–Wallis test. The effect of the biological parameters on the spectrum was evaluated using a multiple linear regression model. A *p*-value < 0.05 was considered a priori to be statistically significant.

## 3. Results

### 3.1. In Vitro Carbamoylation

In vitro carbamoylation of lipoproteins in serum of healthy subjects was demonstrated by agarose gel electrophoresis, which showed a progressive increase in electrophoretic mobility of lipoproteins with increasing KOCN concentrations. The effectiveness of the precipitation reaction was illustrated by the disappearance of the LDL and VLDL fraction on agarose gel electrophoresis. Serum samples with the highest concentrations of KOCN showed a less efficient precipitation, probably due to an altered protein structure, which could interfere with the precipitation process.

**Figure 1:**
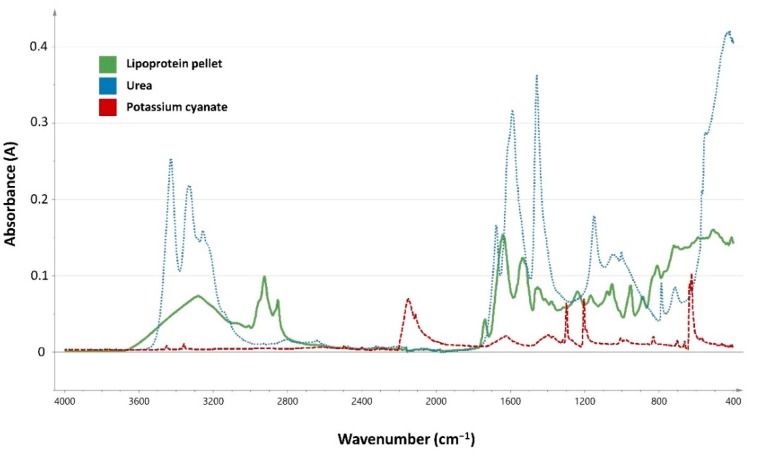
Infrared spectra of precipitated lipoproteins (green), urea (blue) and potassium cyanate (red).

Carbamoylation was further investigated with ATR-FTIR spectroscopy. Figure 1 presents the different IR spectra of the pellet of precipitated lipoproteins, urea, and KOCN. The visual spectrum of urea and KOCN did not interfere with the IR spectra of the precipitated lipoproteins. The between-run and within-run CVs for the detection of the carbonyl band (4.5% and 4.9%), the amide I band (4.9% and 8.4%) and the amide II band (5.8% and 8.1%) were low.

**Figure 2:**
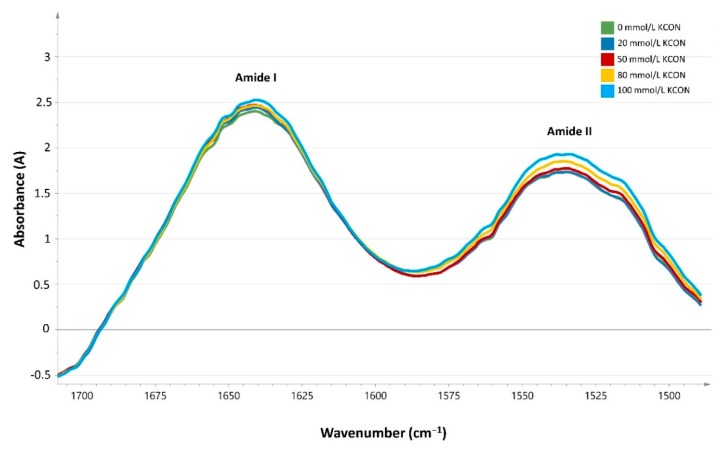
Absorbance spectra of in vitro carbamoylated lipids, adding increasing concentrations of potassium cyanate (0 mmol/L, 20 mmol/L, 50 mmol/L and 100 mmol/L) to serum of healthy subjects.

Using the software package SIMCA 14.1, it was possible to differentiate non-carbamoylated from in vitro carbamoylated non-HDL. The data set was centered, normalized and fitted, and a loading line was formed from the cleaned data. We focused on the fingerprint region (1500–600 cm^−1^) and the amide I and amide II region (1700–1500 cm^−1^). Carbamoylation resulted in a small increase in the amide I band (1714–1589 cm^−1^) of the spectrum (Figure 2). The data set was reduced to the amide I band, normalized and the second derivative was taken before fitting. Moreover, the in vitro experiments showed a diminishing amide II band/amide I area under the curve (AUC) ratio with increasing KOCN concentrations.

In vitro oxidation revealed an increased absorption in the amide I and amide II band (*p* < 0.01). However, the amide II/amide I ratio remained the same before and after in vitro oxidation (ratio 0.55).

### 3.2. In Vivo Samples

Table 1 describes the general characteristics of the healthy subjects and the CKD patients. In the in vivo part of the study, the findings of the in vitro study were compared with the CKD patients’ samples. The same spectral filters were applied and the amide I band was selected. There was a clear distinction between the various groups (healthy subjects, patients with CKD stage 3–5 and CKD 5d patients).

**Figure 3:**
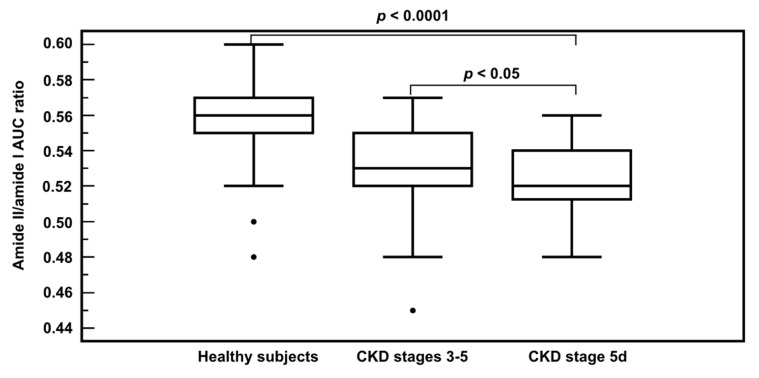
Amide II/amide I AUC ratio among the different study groups.

In addition, a significant difference in the amide II/amide I AUC ratio was observed between the healthy subjects and the CKD groups (*p* < 0.0001) (Figure 3), as well as between the two CKD groups (non-dialysis versus hemodialysis patients). A negative correlation was observed between the amide II/amide I AUC ratio and the serum urea concentration (*r* = −0.63, *p* < 0.0001).

Multiple regression analysis with the amide II/amide I AUC ratio as a dependent variable revealed that the serum urea concentration and the serum apolipoprotein B concentration were the main predictors (Table 2).

## 4. Discussion

In the present study, we have demonstrated for the first time the detection of carbamoylated non-HDL using ATR-FTIR spectroscopy. More specifically, in vitro carbamoylation of non-HDL induced structural changes, which were clearly visible in the mid-IR spectrum of the lipid pellets. The amide I band, containing mainly C=O stretching vibrations of protein peptide bonds was identified as the relevant region. In the clinical part of this study, significant differences at the amide I band were observed between healthy subjects, patients with CKD stage 3–5 and hemodialysis patients (CKD stage 5d). The amide I band depends on the secondary structure of the backbone and is, therefore, the amide vibration, which is most commonly used for secondary structure analysis [22]. The amide II mode is the out-of-phase combination of the N−H in plane bend and the C≡N stretching vibration with smaller contributions from the C=O in plane bend and the C≡C and N≡C stretching vibrations. Although the protein secondary structure and frequency correlate less straightforward than for the amide I vibration, the amide II band provides valuable structural information [23]. Previous studies have attributed the amide I band to apolipoproteins [24,25]. The change in the amide I band can be expected as the carbamoylation process alters the protein component of LDL, namely apolipoprotein B [13].

Building on the in vitro model of carbamoylation, we showed that increasing serum KOCN concentrations resulted in a reduced amide II/amide I AUC ratio. These amide bands in the IR spectrum take part in the adding of the carbamoyl group on the amino group of the epsilon-amino group of lysine and the terminal amino groups. The amide II/amide I AUC ratio reflects the observed spectral changes due to carbamoylation. Significant differences of the amide II/amide I AUC ratio were observed between healthy subjects, patients with advanced stages of CKD and hemodialysis patients. As expected, this ratio showed a negative correlation with the serum urea concentration. This result is supported by earlier findings, showing a similar regression coefficient between % carbamoylated albumin and blood urea concentrations in ESRD subjects [26].

As demonstrated in the multiple regression model, age was identified as a minor predictor of the amide II/amide I AUC ratio in comparison with apolipoprotein B and urea. Tissue accumulation of carbamoylated proteins may be considered as a general hallmark of ageing, linking cumulative metabolic alterations and age-related complications. In addition to the association with carbamoylation, many other nonenzymatic posttranslational modifications occur during the biological life of proteins, leading to protein molecular ageing [27].

A limitation of the present study is the fact that we did not perform liquid chromatography tandem-mass spectrometry (LC-MS/MS) to objectify the amount of carbamoylation. This technique has already been used for the detection of carbamoylated albumin [26], but not for carbamoylated non-HDL. However, the relationship between the amide II/amide I AUC ratio and the KOCN concentration, as well as its relationship with the serum urea concentration are very suggestive for the carbamoylation process of non-HDL. A potential confounder could be the effect of diabetes mellitus, as the lysine residues are susceptible to both carbamoylation and glycation by glucose [28]. However, previous work of our research group showed no significant changes in the amide I and amide II band after in vitro glycation of keratins in nail powder. After incubation of nail powders with respectively 1 mL of 0.9% sodium chloride solution, 5% glucose solution and 10% glucose solution, a clear difference in the area under the infrared peak was observed at wavenumber 1047 cm^−2^, a region characterized by a characteristic carbohydrate absorption. This band was used for measuring the degree of keratin glycation. No influence of glycation products was observed on the amide I and amide II bands [29]. In the CKD stage 3-5 group and in the CKD 5d group, there were respectively 29.7% and 31.3% diabetics as compared to 0% in the group of healthy subjects. In addition, also oxidation may modify lipoproteins in a similar way as it has been demonstrated by amino acid analysis that modification of lysine residues occurs during LDL oxidation [30]. However as demonstrated in our in vitro experiments, oxidation had no important influence on the reported results.

## 5. Conclusions

Carbamoylation is involved in the pathogenesis of various diseases (atherosclerosis, kidney diseases, autoimmune diseases, infections, and thrombus formation) and has been identified as an important risk factor for mortality in dialysis patients or in those with accelerated atherogenesis. The development of novel tools to determine the degree of post-translational modification-derived products is a demanding task. At this moment, the majority of potential carbamoylation biomarkers can only be assessed by rather complex analytical methods, hampering their use in clinical practice [2].

In the present study, we have demonstrated that ATR-FTIR spectroscopy is an easy-to-use, reagent-free, and cost-effective method. It is a non-destructive technique, consuming only a small amount of sample [31]. Spectral changes of non-HDL were observed depending on a declining kidney function. So, ATR-FTIR can be regarded as a new method for identification of carbamoylated non-HDL in CKD patients.

## Figures and Tables

**Table 1:** General characteristics of the healthy subjects and the chronic kidney disease patients.

	Healthy Subjects	CKD Stage 3, 4 or 5	CKD Stage 5d	*p*
N	45	37	47	
Median age (years)	28 (24–33)	70 (56–75)	67 (56–75)	<0.0001
% diabetes mellitus	0	30	32	
Urea (mmol/L)	8.9 (7.8–9.9)	25.0 (18.7–37.5)	32.8 (28.5–40.1)	<0.0001
Creatinine (µmol/L)	72.5 (62.8–80.0)	160.9 (138.6–243.8)	627.6 (474.7–774.4)	<0.0001
eGFR (mL/min/1.73 m^2^)	>90	31.0 ± 13.6	<15	<0.0001
Albumin (g/L)	47.3 (45.0–50.0)	42.2 (40.3–44.5)	40.0 (36.2–42.9)	<0.0001
Total cholesterol (mmol/L)	4.8 (4.3–5.5)	4.7 (3.7–5.5)	4.3 (3.7–5.8)	NS
HDL cholesterol (mmol/L)	1.6 (1.3–2.0)	1.3 (1.0–1.6)	1.1 (0.9–1.5)	<0.0001
LDL cholesterol (mmol/L)	2.7 (2.3–3.1)	2.3 (1.8–3.1)	2.4 (1.7–3.2)	NS
Triglycerides (mmol/L)	1.0 (0.8–1.3)	1.7 (1.1–2.3)	1.4 (1.1–2.4)	=0.0002
Apolipoprotein B (g/L)	0.8 (0.7–1.0)	0.8 (0.7–1.0)	0.8 (0.7–0.9)	NS

NS = not significant.

**Table 2:** Multiple regression model with the amide II/amide I area under the curves (AUC) ratio as dependent variable.

	Variable	β (Standard Error)	*p*
Amide II/amide I AUC ratio, *r*^2^ = 0.54, *p* < 0.001	Triglycerides (mmol/L)	−0.001819 (0.001367)	0.1858
	Apolipoprotein B (g/L)	−0.0306 (0.006277)	<0.0001
	Creatinine (µmol/L)	−0.00001067 (0.000006989)	0.1293
	Urea (mmol/L)	−0.0006622 (0.0001436)	<0.0001
	Age (years)	−0.0002128 (0.00008219)	0.0108
